# MEF2D在肺腺癌中的表达及与预后的相关性分析

**DOI:** 10.3779/j.issn.1009-3419.2023.102.25

**Published:** 2023-07-20

**Authors:** Guangbin YE, Zhongwei ZHANG, Yanli LI, Li GAO, Wei HUANG, Bo LING

**Affiliations:** ^1^533000 百色，右江民族医学院基础医学院; ^1^School of Basic Medical Sciences, Youjiang Medical University for Nationalities, Baise 533000, China; ^2^533000 百色，药学院; ^2^School of Pharmacy, Youjiang Medical University for Nationalities, Baise 533000, China; ^3^401121 重庆，重庆市人民医院心胸外科; ^3^Department of Cardiothoracic Surgery, Chongqing People's Hospital, Chongqing 401121, China

**Keywords:** 肺肿瘤, MEF2D, 预后, 临床特征, Lung neoplasms, MEF2D, Prognosis, Clinical features

## Abstract

**背景与目的** 肌细胞增强因子2D（myocyte enhancer factor 2D, MEF2D）可以通过调控癌基因的转录参与肿瘤病变的进程。前期研究证实，MEF2D通过促进NUSAP1的转录，提高肺腺癌细胞A549和H1299的增殖和转移能力。本研究旨在探讨肺腺癌组织中MEF2D的表达水平及其临床意义。**方法** 收集肺腺癌患者199例，采用免疫组化染色检测癌组织和癌旁组织中MEF2D的表达水平；整理患者病例资料和随访资料，研究MEF2D表达水平、临床指标和预后三者的相关性。**结果** 肺腺癌患者中，癌组织MEF2D高表达率明显高于癌旁组织（P<0.05）。免疫组化结果分析发现，肺腺癌患者的癌组织中MEF2D表达水平与肿瘤分化程度、N分期、M分期和肺内转移具有相关性（P<0.05）。Kaplan-Meier分析证实，MEF2D低表达肺腺癌患者的预后优于高表达者（P<0.05）。Cox多因素分析表明，MEF2D表达水平、M分期、N分期和骨转移是影响肺腺癌患者预后的独立危险因素。**结论** MEF2D表达水平与肺腺癌的转移等临床特征关系密切，可作为患者预后的独立危险因素，可能是肺腺癌诊治的新靶点。

## Abstract (en)

**Background and objective** Myocyte enhancer factor 2D (MEF2D) can participate in the process of tumor lesions by regulating the transcription of oncogenes. In a previous study, MEF2D was demonstrated to enhance the proliferation and metastasis of lung adenocarcinoma cells A549 and H1299 by promoting the transcription of NUSAP1. The research aimed to explore the expression level and clinical significance of MEF2D in lung adenocarcinoma. **Methods** A total of 199 patients with lung adenocarcinoma were collected. Immunohistochemical staining was used to detect MEF2D expression levels in cancer and adjacent tissues. After the clinical and follow-up data were collated, the correlation between MEF2D expression level and clinical characteristics and prognosis of the patients was analyzed. **Results** In the lung adenocarcinoma, the high expression rate of MEF2D in cancer tissues was significantly higher than that in adjacent tissues (P<0.05). According to immunohistochemical score, MEF2D expression level in lung adenocarcinoma tissues was correlated with tumor differentiation, N stage, M stage and intrapulmonary metastasis (P<0.05). Kaplan-Meier analysis showed that patients with low MEF2D expression had significantly better prognosis than patients with high MEF2D expression (P<0.05). Cox multivariate analysis showed that MEF2D expression level, M stage, N stage and bone metastasis of lung cancer were independent risk factors for prognosis of lung adenocarcinoma patients. **Conclussion** MEF2D expression level is closely related to the metastasis of lung adenocarcinoma and other clinical characteristics, and can be used as an independent risk factor for the prognosis of patients with lung adenocarcinoma, which has the potential to be developed as a clinical diagnosis and treatment target of lung adenocarcinoma.

国家癌症中心发布的最新一期的全国癌症统计数据显示肺癌是我国男性发病率和死亡率最高的恶性肿瘤。肺腺癌是肺癌中常见的类型之一（占31.5%），同时也是非小细胞肺癌的重要亚型（占50%）^[1,2]^。最新的流行病理学研究^[3]^表明，非小细胞肺癌的两个主流亚型中，肺腺癌的发病率逐年增高，而肺鳞癌的发病率则呈现下降趋势。肺腺癌具有高发病率和高死亡率的特点，其核心原因是早期筛查和预后指标不明确导致其高转移率和高复发率^[4,5]^。临床中肺腺癌组织病理学检测指标，诸如细胞角蛋白7（cytokeratin 7, CK7）、甲状腺转录因子1（thyroid transcription factor 1, TTF1）、天冬氨酸蛋白酶A（novel aspartie proteinase A, Napsin A）等，在病理诊断的准确性和特异性中仍然存在一定的局限性^[6,7]^。我们前期的研究^[8]^证实肌细胞增强因子2D（myocyte enhancer factor 2D, MEF2D）可促进NUSAP1的转录，增强非小细胞肺癌细胞A549和H1299的增殖和转移能力。MEF2D是非小细胞肺癌化疗中的协同效应分子，MEF2D干扰后可增强顺铂对A549细胞的敏感性，其核心机制源于MEF2D对细胞周期蛋白Cyclin D1和细胞凋亡相关蛋白Caspase-3的调控作用^[9]^。尽管明确了MEF2D在非小细胞肺癌进程中的调控作用，但肺腺癌患者中MEF2D的表达规律和预后特点仍然不清楚。因此，深入分析MEF2D在肺腺癌中的表达，并阐明其与临床指标和预后规律之间的相关性，或可为肺腺癌的早期筛查和预后诊断提供潜在性的分子靶点。

## 1 资料与方法

### 1.1 研究对象

收集右江民族医学院附属医院、重庆市人民医院收治的肺腺癌患者（2010至2016年）199例。患者均通过影像学和病理检测进行确诊，且均接受了手术治疗，切除的肺腺癌组织和癌旁组织均进行石蜡包埋处理。在入组的患者中，手术前均未进行放化疗，均未出现多原发癌，围手术期内均未出现严重的并发症，影像学确定分期情况，所有患者的病例资料齐全且经过严格的随访，随访时间到2020年8月。本研究经过了右江民族医学院（批准号：2019102001）和重庆市人民医院（批准号：2015082104）的批准，并获得了患者及家属的知情同意。

所有入组患者中男性86例，女性113例；≥50岁174例，<50岁25例；依据肿瘤原发部位分组，肺上叶80例，肺下叶100例，肺中叶10例，不明位置9例；肿瘤偏侧性分组，左侧80例，右侧119例；肿瘤分化程度分组，高分化97例，中分化75例，低分化27例；参照美国癌症联合委员会（American Joint Committee on Cancer, AJCC）分期2010年第10版进行肿瘤原发灶-淋巴结-转移（tumor-node-metastasis, TNM）分期，T分期中T1期81例，T2期53例，T3期41例，T4期24例；M分期中M0期152例，M1期47例；N分期中N0期135例，N1期15例，N2期37例，N3期12例。

### 1.2 免疫组织化学染色检测MEF2D的表达

#### 1.2.1 试剂耗材

抗体：一抗为小鼠单克隆抗体MEF2D（H-11，货号sc-271153，Santa Cruz Biotechnology），二抗为抗小鼠IgG二抗（货号sc-533670，Santa Cruz Biotechnology）。SP免疫组化检测试剂采购于北京博奥森生物技术有限公司。DAB显影液采购于Invitrogen公司。

#### 1.2.2 实验方法

将包埋好的组织样本蜡块切片后，进行免疫组化实验，具体实验操作步骤见参考文献^[5]^。

#### 1.2.3 检测结果判读

免疫组化染色评分方法参考相关文献^[10,11]^，依据免疫组化染色评分分为MEF2D高表达组（≥6分）和MEF2D低表达组（<6分）。

### 1.3 统计学分析

本研究数据使用SPSS 17.0软件进行分析。计量资料用Mean±SD表示，两组之间比较采用t检验。计数资料用率表示，各组样本的比较采用卡方检验。Kaplan-Meier制作生存曲线并用Log-rank检验其差异性。Cox多因素分析肺腺癌预后的独立因素。P<0.05为差异有统计学意义。

## 2 结果

### 2.1 肺腺癌患者组织样本中MEF2D的表达

收集肺腺癌患者组织样本，共得到199例肺腺癌的癌组织样本和188例肺腺癌的癌旁组织样本（其中11例癌旁样本切片不完整故而排除）。MEF2D的表达主要分布在细胞核内和细胞质中。在肺腺癌的癌组织样本中，MEF2D高表达患者126例，MEF2D低表达患者73例。在肺腺癌的癌旁组织样本中，MEF2D高表达患者89例，MEF2D低表达患者99例。肺腺癌患者中，癌组织MEF2D高表达率显著高于癌旁组织（P<0.05）。见图1。

**图1:**
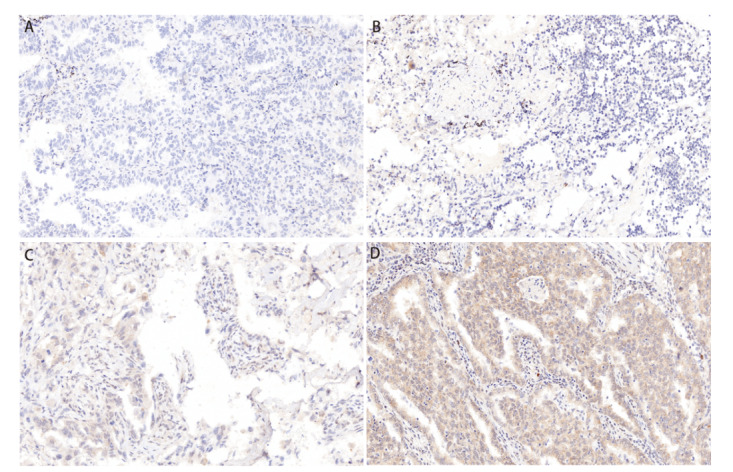
免疫组化染色检测肺腺癌组织中MEF2D的表达（×200）。A：阴性表达（-）；B：弱阳性表达（+）；C：中阳性表达（++）；D：强阳性表达（+++）。

### 2.2 肺腺癌中MEF2D与患者临床特征的相关性

根据肺腺癌的癌组织中MEF2D的免疫组化染色结果和患者的临床资料进行分析，肺腺癌患者的癌组织中MEF2D表达水平与肿瘤分化程度、N分期、M分期和肺内转移具有相关性（P<0.05）；与患者的年龄、性别、肿瘤原发部位、偏侧性、T分期、骨转移、脑转移和肝转移无相关性（P>0.05）。中低分化、N1、N2、N3、M1和肺内转移的肺腺癌患者易出现癌组织中MEF2D的高表达。见表1。

**表1:** MEF2D表达高低水平与肺腺癌临床特征之间的相关性分析[n (%)]

Clinical characteristics	Total (n=199)	MEF2D low expression (n=73)	MEF2D high expression (n=126)	χ^2^	P
Gender				0.026	0.871
Female	113 (56.8)	42 (57.5)	71 (56.3)		
Male	86 (43.2)	31 (42.5)	55 (43.7)		
Age (yr)				0.006	0.940
≤50	25 (12.6)	9 (12.3)	16 (12.7)		
>50	174 (87.4)	64 (87.7)	110 (87.3)		
Primary site				0.618	0.892
Upper lobe	80 (40.2)	27 (37.0)	53 (42.1)		
Middle lobe	10 (5.0)	4 (5.5)	6 (4.8)		
Lower lobe	100 (50.3)	39 (53.4)	61 (48.4)		
NOS	9 (4.5)	3 (4.1)	6 (4.8)		
Grade				7.689	0.021
Well differentiated	97 (48.7)	44 (60.3)	53 (42.1)		
Moderately differentiated	75 (37.7)	24 (32.9)	51 (40.5)		
Poorly differentiated	27 (13.6)	5 (6.8)	22 (17.5)		
Laterality				0.038	0.845
Left-origin of primary	80 (40.2)	30 (41.1)	50 (39.7)		
Right-origin of primary	119 (59.8)	43 (58.9)	76 (60.3)		
T stage				1.76	0.624
T1	81 (40.7)	32 (43.8)	49 (38.9)		
T2	53 (26.6)	16 (21.9)	37 (29.4)		
T3	41 (20.6)	17 (23.3)	24 (19.0)		
T4	24 (12.1)	8 (11.0)	16 (12.7)		
N stage				9.539	0.023
N0	116 (58.3)	52 (71.2)	64 (50.8)		
N1	30 (15.1)	5 (6.8)	25 (19.8)		
N2	38 (19.1)	11 (15.1)	27 (21.4)		
N3	15 (7.5)	5 (6.8)	10 (7.9)		
M stage				7.712	0.005
M0	137 (68.8)	59 (80.8)	78 (61.9)		
M1	62 (31.2)	14 (19.2)	48 (38.1)		
Bone metastasis				0.444	0.505
Yes	11 (5.5)	3 (4.1)	8 (6.3)		
No	188 (94.5)	70 (95.9)	118 (93.7)		
Brain metastasis				0.472	0.492
Yes	6 (3.0)	3 (4.1)	3 (2.4)		
No	193 (97.0)	70 (95.9)	123 (97.6)		
Liver metastasis				3.228	0.072
Yes	10 (5.0)	1 (1.4)	9 (7.1)		
No	189 (95.0)	72 (98.6)	117 (92.9)		
Lung metastasis				5.830	0.016
Yes	33 (16.6)	6 (8.2)	27 (21.4)		
No	166 (83.4)	67 (91.8)	99 (78.6)		

NOS: not otherwise specified; TNM: tumor-node-metastasis.

### 2.3 MEF2D表达水平对肺腺癌预后的影响

Kaplan-Meier分析得出，肺腺癌患者中MEF2D低表达的预后效果显著优于MEF2D高表达（P<0.05）（图2）。对患者的生存率和生存时间进行分析可以得出（表2，表3），MEF2D高表达肺腺癌患者的1、3、5年生存率和平均生存时间均显著低于MEF2D低表达患者（P<0.05）。

**图2:**
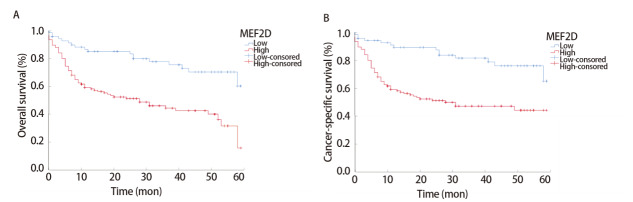
肺腺癌患者组织中MEF2D表达水平与预后的相关性分析。A：总生存期的比较；B：肿瘤特异性生存期的比较。

**表2:** MEF2D表达与总生存期中的生存率、生存时间的相关性分析

Overall survival	1-yr survival rate	3-yr survival rate	5-yr survival rate	Mean survival time (mon)
MEF2D low expression	85.2%	77.9%	60.4%	47.3±2.5
MEF2D high expression	59.2%	44.5%	15.8%	30.3±2.3
χ^2^/t	18.329	16.755	22.684	8.927
P	<0.001	<0.001	<0.001	<0.001

**表3:** MEF2D表达与肿瘤特异性生存期中的生存率、生存时间的相关性分析

Cancer-specific survival	1-yr survival rate	3-yr survival rate	5-yr survival rate	Mean survival time (mon)
MEF2D low expression	89.5%	81.8%	65.3%	49.9±2.3
MEF2D high expression	59.2%	47.4%	44.4%	32.1±2.4
χ^2^/t	15.492	20.968	17.315	9.057
P	<0.001	<0.001	<0.001	<0.001

### 2.4 Cox多因素分析MEF2D与肺腺癌预后的相关性

纳入患者的临床资料和MEF2D水平进行Cox多因素分析得出，MEF2D表达水平、M分期、N分期和骨转移是影响肺腺癌患者预后的独立危险因素。MEF2D高表达、M1期、N1期和骨转移的肺腺癌患者的预后效果较差，见表4。值得注意的是，肺腺癌中分化患者较低分化患者预后差，以及肿瘤原发偏侧性结果存在争议，故剔除。

**表4:** 肺腺癌患者预后的Cox多因素分析

Clinical characteristics	Hazard ratio	95%CI		P
Gender				
Female	1			
Male	0.680	0.391-1.184		0.173
Age (yr)				
≤50	1			
>50	1.851	0.686-4.993		0.224
Primary site				0.840
Upper lobe	1			
Middle lobe	1.417	0.365-5.504		0.615
Lower lobe	0.837	0.480-1.459		0.530
NOS	1.020	0.343-3.036		0.972
Grade				0.023
Well differentiated	1			
Moderately differentiated	2.034	1.126-3.674		0.019
Poorly differentiated	0.913	0.380-2.194		0.839
Laterality				
Left origin of primary	1			
Right origin of primary	0.557	0.314-0.988		0.046
T stage				0.663
T1	1			
T2	0.875	0.434-1.764		0.709
T3	1.377	0.643-2.95		0.411
T4	1.339	0.528-3.391		0.539
N stage				0.041
N0	1			
N1	3.393	1.295-8.889		0.013
N2	1.468	0.544-3.967		0.449
N3	1.864	0.547-6.353		0.320
M stage				
M0	1			
M1	4	1.587-10.082		0.003
Bone metastasis				
No	1			
Yes	2.674	1.137-6.288		0.024
Brain metastasis				
No	1			
Yes	1.119	0.309-4.054		0.865
Liver metastasis				
No	1			
Yes	1.242	0.514-3.001		0.630
Lung metastasis				
No	1			
Yes	1.131	0.521-2.458		0.756
MEF2D expression				
Low	1			
High	2.519	1.318-4.815		0.005

CI: confidence interval.

## 3 讨论

随着医学技术的不断发展，肺腺癌的临床治疗手段也取得了一定的进步，手术、放化疗、分子靶向药物和免疫治疗等多种方法的应用，均取得了良好的临床疗效，并提升了患者的预后效果^[12]^。然而，高转移复发率仍然是制约肺腺癌临床的重要难题。在肿瘤的转移复发中，肿瘤细胞的上皮间质转化（epithelial-mesenchymal transition, EMT）和肿瘤血管新生是核心环节之一^[13]^。转录因子是细胞内信号传递的关键节点，也是肿瘤细胞侵袭转移能力获得的核心调控因子，如Snail、Zeb和Twist等转录因子通过靶向调控E-cadherin、N-cadherin等基因的转录水平，从而参与肿瘤的转移环节^[14]^。然而，鉴于肺腺癌转移环节的复杂性和单一转录因子功能的局限性，开发新的肿瘤转移调控转录因子具有很强的理论意义和临床价值。

转录因子MEF2D被认为是潜在的肿瘤转移的新靶点。在卵巢癌的研究^[15]^中，利用MEF2D siRNA载体干扰SKOV3细胞后，可降低SKOV3细胞增殖和裸鼠成瘤能力。在结直肠癌的研究^[16]^中，MEF2D可提升肿瘤细胞的侵袭能力，同时促进肿瘤细胞EMT表型的形成。同时也有观点^[17]^指出，MEF2D与肿瘤血管新生关系密切，MEF2D可直接诱导血管新生靶点蛋白缺氧诱导因子1α的高表达，从而促进结直肠癌的血管新生。在肝癌的研究^[18]^中，MEF2D持续的高表达将激活转化生长因子-β（transforming growth factor-β, TGF-β）信号通路，同时与Twist结合从而发挥其促进肝癌细胞EMT进程，继而提升肝癌细胞的侵袭和转移能力。而在肝癌免疫逃逸的研究^[19]^中，MEF2D所诱导的肿瘤血管新生是肿瘤细胞产生免疫逃逸的重要因素之一。我们前期研究^[8]^证实MEF2D参与了非小细胞肺癌的转移调控，然而仅局限在肿瘤细胞功能水平。本研究以MEF2D在临床样本中的表达为切入点，整理临床指标和随访资料，利用统计学分析挖掘肺腺癌中MEF2D的表达规律和预后特点。本研究发现MEF2D高表达与肿瘤分期（N/M）、分化水平和肺内转移具有相关性。TNM分期中，N分期可以用于评价肿瘤是否存在淋巴结转移；M分期用于评价肿瘤是否发生转移^[20]^，由此可以得出MEF2D高表达与肺腺癌患者高转移率具有正相关性。

本研究进一步分析了肺腺癌中不同的MEF2D表达水平与预后的关系。Kaplan-Meier分析证实肺腺癌患者（MEF2D高表达）的1、3、5年生存率和平均生存时间均显著低于MEF2D低表达。Cox多因素回归分析证实MEF2D表达水平是肺腺癌患者预后的独立危险因素之一。以上结果提示MEF2D可作为肺腺癌早期筛查和预后分析的潜在靶点，但仍需进一步扩大临床样本量验证和深入挖掘其作用机制。

**Competing interests**

The authors declare that they have no competing interests.

**Author contributions**

Ye GB, Huang W and Ling B conceived and designed the study. Zhang ZW, Li YL and Gao L performed the experiments. Ye GB and Ling B analyzed the data. Huang W and Ling B contributed analysis tools. Ye GB, Huang W and Ling B provided critical inputs on design, analysis and interpretation of the study. All the authors had access to the data. All authors read and approved the final manuscript as submitted.
